# Clinical and Experimental Evidences of Hydrogen Sulfide Involvement in Lead-Induced Hypertension

**DOI:** 10.1155/2018/4627391

**Published:** 2018-03-28

**Authors:** José Sérgio Possomato-Vieira, Victor Hugo Gonçalves-Rizzi, Regina Aparecida do Nascimento, Rodrigo Roldão Wandekin, Mayara Caldeira-Dias, Jessica Sabbatine Chimini, Maria Luiza Santos da Silva, Carlos A. Dias-Junior

**Affiliations:** Department of Pharmacology, Institute of Biosciences, Sao Paulo State University (UNESP), Botucatu, SP, Brazil

## Abstract

Lead- (Pb-) induced hypertension has been shown in humans and experimental animals and cardiovascular effects of hydrogen sulfide (H_2_S) have been reported previously. However, no studies examined involvement of H_2_S in Pb-induced hypertension. We found increases in diastolic blood pressure and mean blood pressure in Pb-intoxicated humans followed by diminished H_2_S plasmatic levels. In order to expand our findings, male Wistar rats were divided into four groups: Saline, Pb, NaHS, and Pb + NaHS. Pb-intoxicated animals received intraperitoneally (i.p.) 1st dose of 8 *μ*g/100 g of Pb acetate and subsequent doses of 0.1 *μ*g/100 g for seven days and sodium hydrosulfide- (NaHS-) treated animals received i.p. NaHS injections (50 *μ*mol/kg/twice daily) for seven days. NaHS treatment blunted increases in systolic blood pressure, increased H_2_S plasmatic levels, and diminished whole-blood lead levels. Treatment with NaHS in Pb-induced hypertension seems to induce a protective role in rat aorta which is dependent on endothelium and seems to promote non-NO-mediated relaxation. Pb-intoxication increased oxidative stress in rats, while treatment with NaHS blunted increases in plasmatic MDA levels and increased antioxidant status of plasma. Therefore, H_2_S pathway may be involved in Pb-induced hypertension and treatment with NaHS exerts antihypertensive effect, promotes non-NO-mediated relaxation, and decreases oxidative stress in rats with Pb-induced hypertension.

## 1. Introduction

Lead (Pb) is an important environmental pollutant that presents hazardous effects for human health. Most of the population must have lead in their organisms due to occupational or environmental exposure [1–3]. A variety of harmful effects may arise from lead intoxication, which include alterations in bone density [4, 5] and cognitive [6, 7] and cardiovascular disorders, such as hypertension [8, 9], and these adverse effects may result from both duration of exposure and blood lead levels [10]. The National Institute for Occupational Safety and Health, USA, stated that reference blood lead levels for adults should be ≥5 *μ*g/dL [11] and lead-induced hypertension has been shown in experimental animals with low blood lead levels (9–37 *μ*g/dL) [9, 12–14]. Multiple mechanisms have been proposed to explain how lead intoxication impairs the cardiovascular system and leads to hypertension. Although these alterations promoted by lead are not completely understood in the early stages of lead exposure, increases in angiotensin II levels resulting from converting enzyme activation [15], increases in matrix metalloproteinases levels/activity [9, 16], increases in cyclooxygenase-derived contractile prostanoids [12], reductions in nitric oxide (NO) bioavailability [9, 13, 17], and increases in reactive oxygen species (ROS) production [18, 19] have been proposed as mechanisms involved in lead-induced hypertension.

Hydrogen sulfide (H_2_S) was formerly known as a pollutant gas with characteristic smell of rotten eggs. Now, there is a large body of literature that indicates H_2_S as a gasotransmitter with important physiological functions [20–25]. H_2_S is enzymatically produced by cystathionine *γ*-lyase (CSE) and cystathionine *β*-synthase (CBS) [26], though other systems have been described as H_2_S producers, such as the 3-mercaptopyruvate sulfurtransferase pathway [27]. H_2_S has been described to participate in several physiological processes, such as neurotransmission, inflammation and immune reactions, gastrointestinal function, cancer development, and cardiovascular responses [20, 28, 29]. Some studies report that H_2_S may exert a vasodilator effect [30, 31] and thus help in the control of vascular tone. Moreover, H_2_S may act as an antioxidant [32, 33]. It has been shown that H_2_S displayed an effective preservation of antioxidants enzymes activity [34–36] and also decreased the levels of different biomarkers of oxidative stress* in vivo* and* in vitro* [34–38]. There are evidences that H_2_S exerts a vasoprotective effect in hypertension and atherosclerosis [39, 40] and that exogenous donors of H_2_S can attenuate vascular dysfunction [41]. Importantly, to our knowledge, no studies have evaluated the role of H_2_S in Pb-induced hypertension.

As consistent vasoconstriction and increases in oxidative stress are well-known hallmarks of hypertension and Pb-induced hypertension has been previously reported, we hypothesized that low-lead-level intoxication causes hypertension and reduces H_2_S plasmatic levels in humans. In order to advance in the knowledge of H_2_S role in Pb-induced hypertension, we suggested that sodium hydrosulfide (NaHS), a donor of H_2_S, blunts the increases in systolic blood pressure (SBP) caused by low-lead-level intoxication in rats and that this beneficial effect on blood pressure may be related to H_2_S antioxidant capacity.

## 2. Materials and Methods

### 2.1. Blood Pressure Measurements and Blood Collection in Human Subjects

Subjects were recruited from the Center for Toxicological Assistance (CEATOX)/UNESP, Botucatu. Written informed consent was obtained from all subjects, and the study was approved by the committee and Institutional Review Board of the Faculdade de Medicina de Botucatu, UNESP (protocol number: 16354513.0.0000.5411). Only male patients were included in this study. Blood pressure measurements were accessed in left arm using an automated device (Z-40, Techline, São Paulo, Brazil). Whole-blood heparin and plasma heparin were collected to perform lead levels analysis and biochemical assays, respectively. Samples were stored at −80°C. Patients with whole-blood lead levels less than 5 *μ*g/dL were included as patients from control group (*n* = 25) and patients with whole-blood lead levels more than 5 *μ*g/dL were included as Pb-intoxicated group (*n* = 20).

### 2.2. Animals and Treatments

Forty male Wistar rats weighing approximately 250 ± 20 g were used in this study. Animals were kept in standard rat cages, maintained under controlled temperature (22°C) on a 12-hour light-dark cycle, and given free access to water and rat chow. All procedures for animal experimentation were approved by Ethics Committee, Institute of Biosciences, Sao Paulo State University, Botucatu (protocol number: 458/2013), which complied with international guidelines of the European Community for the use of experimental animals.

The animals were randomly divided into two lead-exposed groups (Pb and Pb + NaHS; *n* = 12 per group) and two control (non-lead-exposed) groups (Saline and NaHS; *n* = 8 per group) for eight days. Animals exposed to lead were injected intraperitoneally (i.p.) with a first dose of 8 *μ*g/100 g of lead acetate (Pb(C_2_H_3_O_2_)_2_ + 3H_2_O, 100% purity, Merck, USA) and subsequent daily doses of 0.1 *μ*g/100 g to cover daily loss and treatment with NaHS (50 *μ*mol/Kg/twice daily;* Pb + NaHS group*) or vehicle (water;* Pb group*) i.p. for seven days. Animals from control groups (non-lead-exposed) were injected i.p. with a first dose of 8 *μ*g/100 g of sodium acetate (Na(C_2_H_3_O_2_) + 3H_2_O, 99% purity, J.T.Baker, Canada), a subsequent dose of 0.1 *μ*g/100 g to cover daily loss, and treatment with NaHS (50 *μ*mol/Kg/twice daily;* NaHS group*) or vehicle (water;* Saline group*) i.p. for seven days. The protocol of intoxication with lead acetate used in this study was based on previous studies [9, 13, 17].

On the eighth day (i.e., 24 hours after each respective treatment), animals were anesthetized with isoflurane (2–4%) and killed by exsanguination. After thoracotomy, the descending thoracic aortas were removed for vascular experiments studies. Whole-blood samples were collected in tubes containing lyophilized heparin (*Vacutainer *BD, trace metal-free) to access lead concentrations. Blood was also collected in tubes containing ethylenediaminetetraacetic acid (EDTA) (*Vacutainer* Becton-Dickinson, Oxford, UK) and sodium citrate (*Vacutainer* Becton-Dickinson, Oxford, UK) for plasma separation. Plasma was stored at −80°C until biochemical analyses.

### 2.3. Blood Pressure Measurements

SBP (mmHg) was measured using tail-cuff plethysmography (Insight, Ribeirao Preto, Sao Paulo, Brazil). Briefly, conscious rats were first acclimated in a quiet room, conditioned, and restrained for 5–10 minutes in a warm box (Insight, Ribeirao Preto, Sao Paulo, Brazil). Animals were “trained” to the measurements process for 3 days before the beginning of the treatments (data were discarded). SBP was measured, and the mean of three measurements was recorded from day one to day seven of the experimental protocol, as previously described [9, 13]. Blood pressure measurements were performed one hour before the injections of sodium acetate/Pb/NaHS.

### 2.4. Determination of Lead Concentrations in Whole Blood

Pb concentration in whole blood from human patients and rats was determined by graphite furnace atomic absorption spectrometry (GF-AAS; Varian SpectrAA 220) as previously described [9, 13]. Briefly, blood samples were diluted 1 + 49 with a diluent solution containing 0.5% (v/v) double-distilled HNO_3_ 25 *μ*g/l Rh and 0.005% (v/v) Triton® X-100. Calibration was performed against matrix matching. The detection limit of the method was 0.5 *μ*g/L. The whole-blood lead concentrations were expressed in *μ*g/dL.

### 2.5. Determination of H_2_S in Plasma

Plasma obtained from human patients and experimental animals was used for the determination of H_2_S as described previously [42, 43]. In brief, 75 *μ*L of plasma was mixed with 250 *μ*L of zinc acetate 1% (wt/vol) and 425 *μ*L of water. To the mixture, 150 *μ*L of 20 mmol/L of* N*-dimethyl-*p*-phenylenediamine sulfate in HCl 7.2 M and 150 *μ*L of 30 mmol/L of FeCl_3_ in HCl 1.2 M were added. After 10 minutes of incubation at room temperature, 250 *μ*L of 10% trichloroacetic acid was added to remove proteins and the reaction mixture was centrifuged at 12000*g* for 15 minutes. The absorbance of the resulting supernatant (200 *μ*L) was measured at 670 nm with a spectrophotometer (Synergy 4, BioTek, Winooski, VT, USA) in a 96-well plate. The concentration of H_2_S in the solution was calculated against a calibration curve of NaHS (100–3.13 *μ*M).

### 2.6. Vascular Reactivity

Rats' thoracic aortas were dissected in 3-4 mm segments (two rings). One of the rings had its endothelium mechanically removed. The rings were mounted into a 10 mL organ chamber containing Krebs-Henseleit solution (NaCl 130; KCl 4.7; CaCl_2_ 1.6; KH_2_PO_4_ 1.2; MgSO_4_ 1.2; NaHCO_3_ 15; glucose 11.1; in mmol/L) and suspended between two wire hooks; one hook was fixed to a stationary support, and the other hook connected to an isometric force transducer. The Krebs-Henseleit solution was kept at pH 7.4 and 37°C and bubbled continuously with a mixture of 95% O_2_ and 5% CO_2_. Arterial rings were stretched under 1.5 g basal tension and were allowed to equilibrate for 45 min. Changes in aorta tension were recorded using FORT10 isometric force transducers (WPI, USA) connected to Transbridge 4M Transducer Amplifier (WPI, USA) connected to a PC-based MP100 System and analyzed offline using AcqKnowledge version 3.5.7 software (Biopac Systems Inc., USA).

After tissue equilibration, a control contraction to 96 mM of KCl was elicited. Once KCl maximum contraction was reached, tissue was rinsed with Krebs 3 times, 15 minutes each. Then, aorta rings were stimulated with increasing concentrations of phenylephrine (PHE, 10^−10^ to 10^−4 ^M). In order to investigate endothelial function, vascular tissues were precontracted with PHE (10^−6 ^M for intact rings and 3 × 10^−7 ^M for denuded rings); increasing concentrations of acetylcholine (ACh, 10^−9^ to 10^−5 ^M) were added to the bath. To evaluate the participation of endothelium-dependent NO in aortic rings relaxation, concentration-response curves to ACh were obtained in the presence of N*ω*-nitro-L-arginine-methyl ester (L-NAME, 3 × 10^−4 ^M), added in the last 30-minute stabilization period [44, 45]. Concentration-contraction curves were constructed, and the maximal response to PHE was measured. Concentration-effect curves to ACh, with or without L-NAME, were expressed as the % relaxation to PHE contraction. Nonlinear regression (variable slope) of the obtained concentration-effect curves revealed *R*_max⁡_ (maximal response) and pEC50 (negative logarithm of the concentration that evoked 50% of the maximal response).

### 2.7. Cell Culture and Plasma Incubation

Human umbilical vein endothelial cell (HUVEC) line (CRL 2873) was obtained from American Type Culture Collection (ATCC) (Manassas, VA, USA). HUVECs were cultured in DMEM medium (Gibco, CA, USA) supplemented with 10% (v/v) fetal calf serum (FCS) (Gibco), 50 *μ*g/ml penicillin, 50 *μ*g/ml streptomycin, and 0.5 *μ*g/ml amphotericin B (Gibco) at 37°C in 5% CO_2_ incubator. After reaching 80% confluence, HUVECs were resuspended in DMEM medium and replated in 96-well tissue culture plates (Corning), where they were grown to 80% confluence for incubation experiments. Then, the medium was removed and cells were washed twice in PBS. Cells were incubated in medium, without FCS, with 5% (v/v) plasma from rats treated with Saline, NaHS, Pb, and Pb + NaHS for 24 h. Cell viability was performed by MTT assay as described previously [46]. Viability was compared to control (untreated cells, 100% viability). Cell culture supernatant (CCS) was then stored and kept at −80°C for posterior analysis.

### 2.8. Determination of Nitrite in Culture Supernatant and Plasmatic Nitrite/Nitrate (Total NO*x*)

Nitrite levels were assessed in HUVECs culture supernatant in duplicate using Griess reagents [47]. Briefly, 50 *μ*L of samples was incubated with 50 *μ*L of 1% sulfanilamide solution in 5% phosphoric acid for 10 minutes protected from light. Then, 50 *μ*L of 0.1% N-(1-Naphthyl)-ethylenediamine dihydrochloride solution was added followed by 10-minute incubation. Plate was read in spectrophotometer (Synergy 4, BioTek, Winooski, VT) at 540 nm. A standard curve was generated by incubation of nitrite solutions (0.46–29.5 *μ*mol/L) with the previous reagents.

Plasma total NO*x* concentrations were determined using Griess reagents followed by reduction of nitrous species with vanadium chloride III [48]. Briefly, before addition of Griess reagents, plasma was incubated with 100 *μ*L of saturated solution of vanadium chloride III for 3 hours at 37°C with agitation. Absorbance at 535 nm was read at spectrophotometer (Synergy 4, BioTek, Winooski, VT) and NO*x* concentrations in plasma were calculated using a standard curve of sodium nitrite (1.56–100 *μ*M). Nitrite levels in HUVECs supernatant and total NO*x* levels in plasma were expressed in *μ*mol/L.

### 2.9. Determination of Lipid Peroxidation

Lipid peroxidation was determined in rats' plasma through the formation of malondialdehyde (MDA). MDA reacts with 2-thiobarbituric acid (TBA) and produces a colorimetric reaction that is measured by spectrophotometer at wavelength of 532 nm [49]. In test tubes, a reaction mixture containing 100 *μ*L of distilled water, 50 *μ*L of 8.1% sodium dodecyl sulfate (SDS), 100 *μ*L of plasma samples, 375 *μ*L of acetic acid 20%, and 375 *μ*L of TBA 0.8% was incubated in water bath at 95°C for one hour and subsequently centrifuged at 4000 rpm for 10 minutes. Standard curve was prepared in a similar manner, replacing samples with 25 *μ*L of known concentrations of MDA. Plasmatic TBA reactive species (TBARS) were calculated against a standard curve of MDA (20–320 nmol). Results were expressed as plasma MDA levels in nmol/mL.

### 2.10. Evaluation of the Plasmatic Antioxidant Status

Direct reductions of MTT (3-(4,5-dimethylthiazol-2-yl)-2,5-diphenyltetrazolium bromide, Sigma, St Louis, MO, USA) were assessed to determine the antioxidant status of rats' plasma, as previously described [50]. Briefly, 12.5 *μ*L of dye solution (5 mg/mL in PBS) was mixed with 100 *μ*L of plasma and final volume was adjusted to 200 *μ*L with PBS. Reaction mixture was incubated for 60 min at 37°C and then the reaction was terminated by the addition of 750 *μ*L of 0.04 M hydrochloric acid in isopropanol. Tubes were centrifuged for 10 min at 1000*g* and the absorbance of the collected supernatant was measured at 570 nm. Results are expressed as % of control group (Saline group taken as 100% of antioxidant status).

### 2.11. Data Analysis and Statistics

Statistical analyses were performed using GraphPad Prism® 6.0 software (San Diego, CA). The results are expressed as means ± SEM. For human parameters, comparisons were made using Student's *t*-test. Comparisons between animal groups were assessed by one-way analysis of variance (ANOVA) followed by Tukey's test. For vascular reactivity experiments, individual concentration-contraction or concentration-relaxation curves were constructed; sigmoidal curves were fitted to the data using the least square method, and the comparisons among *R*_max⁡_ and pEC_50_ values were determined by ANOVA followed by Tukey's test. A *P* < 0.05 was considered significant.

## 3. Results

### 3.1. Pb-Intoxication Is Related to Increased Blood Pressure and Decreased H_2_S Levels in Human Subjects

**Figure 1:**
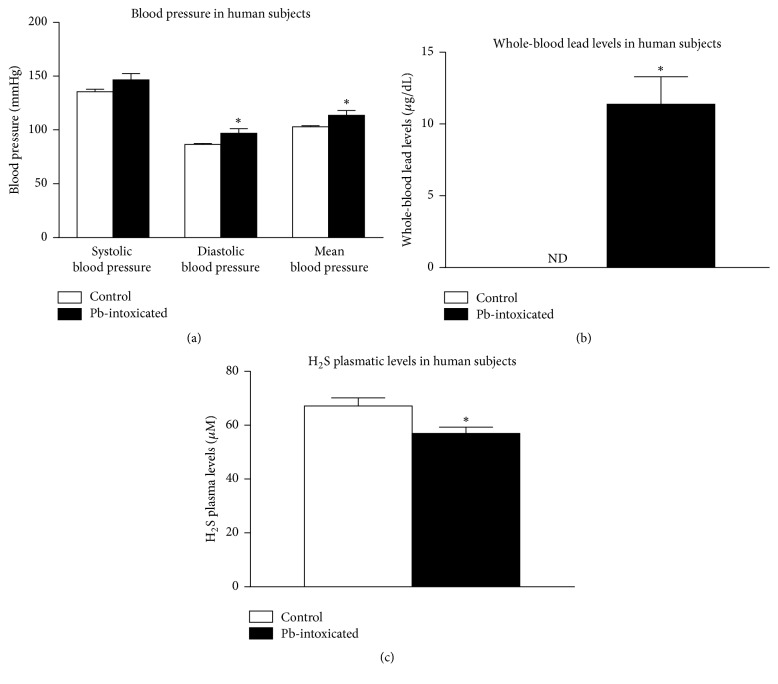
Blood pressure parameters and biochemical analysis in humans. (a) Systolic, diastolic, and mean blood pressure of control and Pb-contaminated patients. (b) Whole-blood lead levels of control and Pb-contaminated patients. (c) H_2_S plasmatic levels of control and Pb-contaminated patients. Values represent mean ± SEM. *n* = 20–25. “ND” means nondetectable. ^*∗*^*P* < 0.05 versus control group.

Hypertension has been reported previously as a consequence of Pb-intoxication; however, no linkage has been made with hydrogen sulfide levels. In our study, there were no significant differences in SBP in humans intoxicated with Pb (146 ± 5 mmHg) compared to control subjects (135 ± 3 mmHg) (*P* = 0.08, Figure 1(a)). However, diastolic blood pressure and mean blood pressure from patients intoxicated with Pb (96 ± 4 and 113 ± 4 mmHg, resp.) were higher than diastolic blood pressure and mean blood pressure of control subjects (86 ± 1 and 102 ± 2 mmHg, resp.) (*P* = 0.02 and *P* = 0.02, resp., Figure 1(a)). The values of whole-blood lead levels in Pb-intoxicated subjects were 11.38 ± 1.92 *μ*g/dL and whole-blood lead levels in control group were below the limit of detection of the technique (*P* = 0.0002, Figure 1(b)). Plasmatic H_2_S levels in control group were 67.15 ± 2.99 *μ*M, whereas subjects from Pb-intoxicated group presented lower H_2_S plasmatic levels (56.99 ± 2.28 *μ*M) (*P* = 0.01, Figure 1(c)).

### 3.2. NaHS Treatment Blunts Pb-Induced Hypertension in Rats and Reduces Whole-Blood Lead Levels

**Figure 2:**
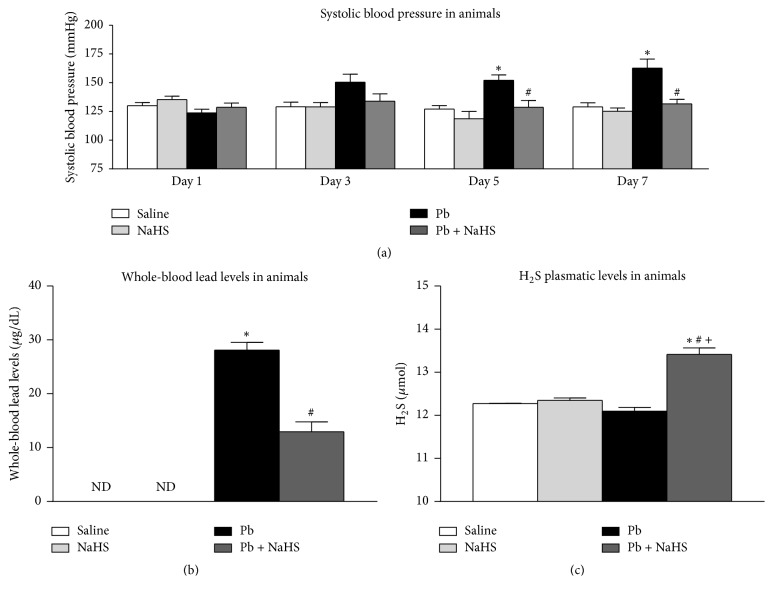
Blood pressure parameters and biochemical analysis in rats among four different groups: Saline (non-Pb-exposed), NaHS (non-Pb-exposed + NaHS), Pb (Pb-exposed), and Pb + NaHS (Pb-exposed + NaHS). (a) Systolic blood pressure measured with tail-cuff plethysmography on days 1, 3, 5, and 7 of experimental protocol. (b) Whole-blood lead levels measured after 7 days of Pb exposition. (c) H_2_S plasmatic levels. Values represent mean ± SEM. *n* = 6–12. ^*∗*^*P* < 0.05 versus Saline group; ^#^*P* < 0.05 versus Pb group; ^+^*P* < 0.05 versus NaHS group.

In order to better understand the involvement of H_2_S in Pb-induced hypertension, we performed experiments in rats. There were no significant differences in SBP among four groups in day one (123 to 132 ± 3 mmHg, Figure 2(a)) and day three (129 to 150 ± 7 mmHg, Figure 2(a)). Animals from Pb group showed an increase in SBP versus Saline on days five (152 ± 5 versus 127 ± 3 mmHg, *P* = 0.007, Figure 2(a)) and seven (163 ± 8 versus 129 ± 3 mmHg, *P* = 0.001, Figure 2(a)). Moreover, this increase was blunted by treatment with NaHS on days five (128 ± 6 mmHg, *P* = 0.007, Figure 2(a)) and seven (131 ± 4 mmHg, *P* = 0.0007, Figure 2(a)).

No lead was detected in whole blood of animals from groups Saline and NaHS in the end of seven days of experimental protocol; however, rats from Pb group presented whole-blood lead levels of 28.12 ± 1.45 *μ*g/dL and an approximately 2-fold decrease was found in animals from Pb + NaHS group (12.96 ± 1.81 *μ*g/dL, *P* = 0.001, Figure 2(b)). H_2_S levels in plasma were significantly higher in group Pb + NaHS (13.42 ± 0.15 *μ*mol) versus Saline (*P* = 0.0001), NaHS (*P* = 0.0001), and Pb groups (*P* = 0.0001) (12.27 ± 0.01, 12.35 ± 0.05, and 12.10 ± 0.08 *μ*mol, resp., Figure 2(c)).

### 3.3. Protective Effect of NaHS Treatment on Vascular PHE-Induced Contraction in Hypertensive Animals Is Dependent on Endothelium

**Figure 3:**
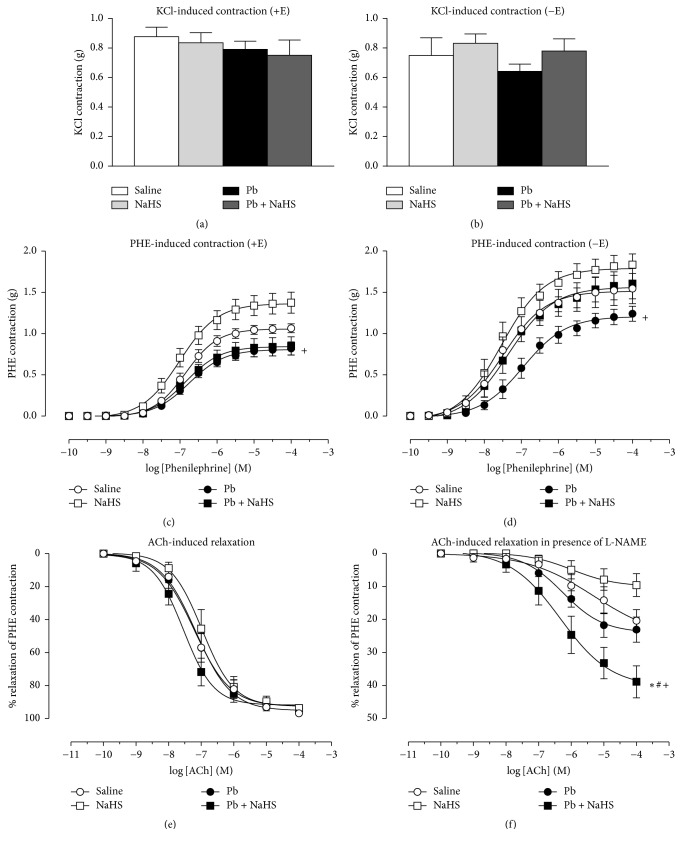
Vascular reactivity of thoracic aorta rings with (+E) or without (−E) endothelium from animals of four different groups: Saline (non-Pb-exposed), NaHS (non-Pb-exposed + NaHS); Pb (Pb-exposed), and Pb + NaHS (Pb-exposed + NaHS). ((a) and (b)) KCl-induced contraction. ((c) and (d)) PHE-induced contraction. (e) ACh-induced relaxation. (f) ACh-induced relaxation in presence of L-NAME. Values represent mean ± SEM. *n* = 5–7. ^+^*P* < 0.05 versus NaHS group; ^*∗*^*P* < 0.05 versus Saline group; ^#^*P* < 0.05 versus Pb group.

Vascular reactivity experiments were performed to assess direct vascular responses among the four different animal groups. KCl-induced contraction was not different between the experimental groups in aortic rings with (+E) or without (−E) endothelium (Figures 3(a)-3(b), Table 1). No differences in maximum response (*R*_max⁡_) to PHE-induced contraction were observed between Saline, Pb, and Pb + NaHS groups in endothelium intact rings; however, greater *R*_max⁡_ was reached in NaHS versus Pb (*P* = 0.0035) and Pb + NaHS (*P* = 0.0077) groups (Figure 3(c), Table 1). No differences were observed in pEC_50_ among the four experimental groups. Denuded rings (−E) presented higher *R*_max⁡_ compared to *R*_max⁡_ of respective group with intact ring (+E) (*P* = 0.0001) and an increase in pEC_50_  (*P* = 0.0001) was also observed except in Pb group (Table 1). Removal of endothelium caused a higher PHE-induced contraction in NaHS versus Pb group (*P* = 0.0374, Figure 3(d), Table 1) and no differences in *R*_max⁡_ were observed among the other groups. No differences were observed in pEC_50_ among the experimental groups with denuded rings (Table 1).

### 3.4. Treatment with NaHS in Pb-Induced Hypertension Elicits an ACh-Induced Relaxation That Is Non-NO-Mediated

Relaxant responses evoked by ACh (that trigger NO release from endothelial cells) were tested in endothelium intact (+E) rings precontracted with PHE. No differences in *R*_max⁡_ and pEC_50_ to ACh-induced relaxation were observed among all four groups (Figure 3(e), Table 1). Blockade of nitric oxide synthase (NOS) using L-NAME caused a decrease in relaxation in Saline, NaHS, and Pb groups (around 20% of relaxation); however, maximum relaxation was greater in rings from Pb + NaHS group compared to the other groups (around 40% of relaxation) (*P* = 0.004, Figure 3(f), Table 1).

**Figure 4:**
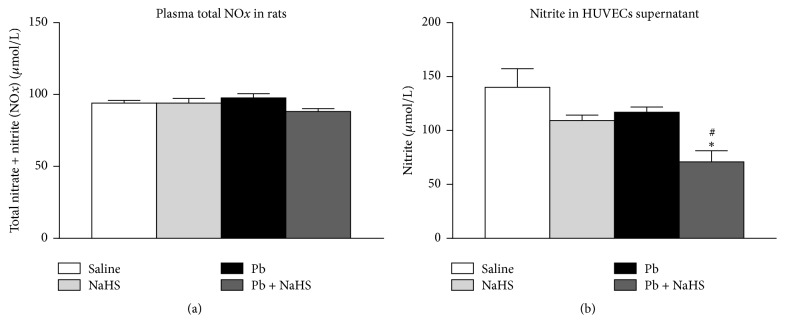
Nitric oxide availability in plasma and cell culture supernatant: Saline (non-Pb-exposed), NaHS (non-Pb-exposed + NaHS), Pb (Pb-exposed), and Pb + NaHS (Pb-exposed + NaHS). (a) Total plasmatic NO*x*. (b) Nitrite levels assessed in supernatant of HUVECs incubated with 5% (v/v) of plasma from animals of four different groups. Values represent mean ± SEM. *n* = 6–12. ^*∗*^*P* < 0.05 versus Saline group; ^#^*P* < 0.05 versus Pb group.

ACh elicits a vasodilatory effect that is dependent on endothelial NO. As we observed relaxation in Pb + NaHS group even in the presence of L-NAME, we sought to investigate the effect of NaHS treatment in NO production. Plasmatic total NO*x* was not different between animals from experimental groups (94.08 ± 1.82, 94.04 ± 3.27, 97.69 ± 2.91, and 88.18 ± 2.03 *μ*mol/L in groups Saline, NaHS, Pb, and Pb + NaHS, resp., Figure 4(a)). Experiments were performed to evaluate the production of NO directly by endothelial cells; therefore, HUVECs were incubated with plasma from animals of the different groups. Surprisingly, nitrite levels were decreased in supernatant of HUVECs incubated with plasma from Pb + NaHS group (70.96 ± 10.37 *μ*mol/L) compared with Saline (140.10 ± 17.14 *μ*mol/L, *P* = 0.0020) and Pb (116.90 ± 4.92 *μ*mol/L, *P* = 0.0261) groups (Figure 4(b)).

### 3.5. Treatment with NaHS Reduces Oxidative Stress in Pb-Induced Hypertension

**Figure 5:**
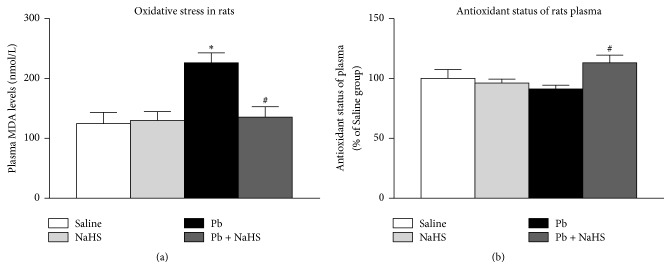
Oxidative stress parameters. Saline (non-Pb-exposed), NaHS (non-Pb-exposed + NaHS), Pb (Pb-exposed), and Pb + NaHS (Pb-exposed + NaHS). (a) MDA levels in plasma. (b) Antioxidant status of plasma as determined by MTT assay. Values represent mean ± SEM. *n* = 6–12. ^*∗*^*P* < 0.05 versus Saline group; ^#^*P* < 0.05 versus Pb group.

Plasmatic MDA levels were elevated in animals exposed to Pb (226.10 ± 16.57 nmol/L) compared with animals from Saline (*P* = 0.0014) and NaHS (*P* = 0.0036) groups (124.70 ± 18.56 and 130.00 ± 14.62 nmol/L, resp.); however, treatment of animals exposed to Pb with NaHS blunted the increase in plasmatic MDA (135.40 ± 17.61 nmol/L in Pb + NaHS group, *P* = 0.0025, Figure 5(a)). Antioxidant status of plasma was not different between Saline and Pb groups (104.60 ± 6.67 and 91.14 ± 3.22%, resp.) (Figure 5(b)). NaHS alone did not alter antioxidant status of plasma (96.20 ± 3.25% in NaHS group); however, plasma from animals of Pb + NaHS group showed an increase in the antioxidant status of plasma (113.10 ± 6.40%) compared to Pb group (*P* = 0.020, Figure 5(b)).

## 4. Discussion

In our study, we observed an increase in diastolic and mean blood pressure in humans intoxicated with Pb and this was related to decreased plasmatic levels of H_2_S. We confirmed that acute exposition to low lead levels promoted an increase in SBP measured by tail cuff plethysmography in awaken rats. Moreover, we showed that treatment with H_2_S donor, NaHS, was able to blunt the increases in SBP promoted by the acute exposition to low lead levels and that these effects of NaHS on blood pressure may be related to H_2_S actions in vascular responses and H_2_S antioxidant capacity.

We observed that patients intoxicated with Pb presented SBP similar to control patients. However, we detected an increase in diastolic and mean blood pressure in Pb-intoxicated patients, which is in accordance with previous reports in literature showing increases in blood pressure following Pb-intoxication in humans [8, 51–55]. As H_2_S has been shown to have several actions in cardiovascular system, we investigated the levels of H_2_S in plasma from patients and found decreased plasmatic H_2_S levels in patients from Pb-intoxicated group. This result is in accordance with previous studies showing that decreased levels of H_2_S are related to hypertension [42, 56–58] and this observation leads us to the interest in understanding the role of this gaseous mediator in Pb-induced hypertension.

In the present study, following intoxication with lead acetate, rats developed higher SBP from exposition day five to day seven, whereas treatment with NaHS blunted this increase in blood pressure. In accordance, we detected the presence of lead in whole blood in the animals from the Pb group. Interestingly, we observed an approximately 2-fold decrease in whole-blood lead levels in animals treated with NaHS (Pb + NaHS group) compared with animals from Pb group. In fact, the reaction of H_2_S with lead acetate generates lead (II) sulfide (PbS) [59].

PbS is a solid, dark color, almost insoluble compound. Indeed, PbS is insoluble and is a very stable compound at blood's pH [60]. Thus, the interaction of H_2_S with lead acetate and consequent formation of PbS in animals from Pb + NaHS group may explain the lower whole-blood lead levels detected in our study. Accordingly, previous studies in rats fed with different compounds of lead showed that animals receiving PbS presented diminished whole-blood lead levels when compared with rats receiving different Pb-based compounds, such as lead acetate and lead oxide [60].

One may consider that the nonobserved increase in SBP in animals from Pb + NaHS group may be due to the lower whole-blood Pb levels. Although the diminished Pb levels in Pb + NaHS group may partially contribute to the attenuation in the increases of SBP, these animals presented whole-blood Pb levels of 12.96 ± 1.81 *μ*g/dL. In addition, it has been shown that whole-blood lead levels below or very close to those found in our study are related to increases in blood pressure. Previous studies from our group showed that animals with 9 ± 1 *μ*g/dL [9] and 11.5 ± 1.2 *μ*g/dL [13] presented an increase in SBP and other groups also reported an increase in blood pressure of rats with whole-blood lead levels of 12 ± 1.34 *μ*g/dL [12] and 9.98 ± 1.70 *μ*g/dL [18]. Therefore, a mechanistic action of H_2_S must be involved in the decreases of blood pressure promoted by NaHS in Pb-induced hypertension.

Interestingly, followed by the reductions in whole-blood lead levels, H_2_S in plasma was increased in Pb + NaHS group. As H_2_S and lead acetate may interact and the PbS generated is expected to be eliminated from the organism, one would inquire that H_2_S should be lower in Pb + NaHS group. However, it has been demonstrated that NaHS may induce the formation of polysulfides (H_2_S_n_, *n* > 1) [61] and that either endogenous or exogenous H_2_S may generate polysulfides through its interaction with ROS, such as superoxide anion [62–64].

Lead-intoxicated animals (Pb group) presented increases in oxidative stress, which was reduced in Pb + NaHS group. Reductions in oxidative stress in animals of Pb + NaHS group may be related to the reaction of exogenous H_2_S with ROS. Also, recent studies suggest that effects initially described for H_2_S may rely (partially or totally) on polysulfides [65]; therefore, polysulfides generated in Pb + NaHS animals may be responsible for the beneficial effects observed in our study. Importantly, the method used in our study to measure H_2_S, the methyl blue formation method, may not distinguish between H_2_S itself and polysulfide compounds. Thus, increased levels of H_2_S shown in Figure 2(c) may represent polysulfides formation.

In order to understand the vascular effects promoted by lead intoxication and the NaHS treatment, vascular reactivity experiments have been performed. No differences were observed in KCl-induced contraction between groups with (+E) or without (−E) endothelium. However, greater contraction to PHE was reached by aortic rings from animals treated with NaHS when compared to both groups receiving Pb (Pb and Pb + NaHS group). While the findings on KCl-induced contraction do not point to increased vascular Ca^2+^ influx through voltage-gated Ca^2+^ channels [44, 66], PHE may also activate Ca^2+^ influx through receptor and store-operated Ca^2+^ channels [44, 66]; thus a greater influx of Ca^2+^ induced by changes promoted by the NaHS treatment must be considered. Also, the increases in contractile responses in NaHS group could be explained by changes in endothelium-dependent relaxation pathways. It was demonstrated that, in low doses, NaHS elicited a vasoconstriction in rats' aortas and mixing NaHS with NO donors inhibited the vasorelaxant effect of NO, both* in vitro* and* in vivo *[67]. Since the procontractile effects of H_2_S were prevented by removing the endothelium or inhibition of eNOS, it has been suggested that H_2_S acted to remove the basal vasorelaxatory influence of NO to produce contraction. Further, these authors showed that H_2_S and NO may interact to generate an inactive nitrosothiol product [68].

Removal of endothelium promoted increases in *R*_max⁡_ and pEC_50_ (*P* < 0.05, Table 1); however, no differences were observed in pEC_50_ between intact and denuded rings from Pb group (*P* > 0.05, Table 1). This indicates that presence of endothelium and, consequently, the protective factors released by endothelial cells were not able to counterbalance the vasoconstrictor effect of PHE in Pb group, which may suggest the installation of some endothelial damage after seven days of exposure to low lead levels.

Rings from Pb and Pb + NaHS groups presented the lower mean values of contraction, and although these values were not significantly different from Saline group, they reached statistical significance compared to NaHS group. Interestingly, Pb + NaHS aortic rings presented decreased *R*_max⁡_ when compared to NaHS group only in rings with intact endothelium, whereas removal of endothelial cells abolished this diminished contraction. Since removal of endothelium abolished this difference in contraction between Pb + NaHS group and NaHS group, we suggest that NaHS treatment may exert a protective effect that is dependent on endothelium only in the presence of hypertension. Vascular effects of H_2_S are not fully comprehended and both contraction [58, 69, 70] and relaxation [58, 67, 71–73] have been shown to occur as an effect of H_2_S. It has been shown before that removal of endothelium [73] or inhibition of NO production [74] reduced relaxant responses to H_2_S; this supports our findings in which removal of endothelium abolished this protective effect induced by NaHS treatment in hypertensive animals.

Although relaxation induced by interactions of H_2_S with NO has been described previously [31, 71, 75, 76], in our hands, following Pb exposition and NaHS treatment, no differences in endothelial NO-dependent relaxation promoted by ACh were observed. However, when relaxation was triggered by ACh in the presence of NOS inhibitor L-NAME, a relaxation around 40% was reached in aortic rings from animals of Pb + NaHS group. Previous studies showed that H_2_S may increase expression and activity of eNOS [77, 78], thus producing more NO, and this excessive NO could be responsible for relaxation observed in the presence of L-NAME. However, we did not observe any alteration in plasmatic total NO*x* from animals of different groups; and interesting data was observed with nitrite measured in HUVECs' culture supernatant. Following incubation with plasma from different animal groups, we observed that cells treated with plasma from Pb + NaHS group presented lower nitrite levels compared to cells treated with plasma from Saline and Pb groups. These results support our observation in vascular reactivity experiments where relaxation in Pb + NaHS group may be triggered in an NO-independent way. Since NO-independent relaxation was reached in vessels from Pb + NaHS group and not in vessels from animals treated only with NaHS, we speculate that only in presence of hypertension, which may trigger several defensive mechanisms in the organism, does H_2_S promote relaxation independently from NO presence and strongly recommend future studies in the field.

As interaction with NO seems not to be involved in the beneficial effects of H_2_S in Pb-induced hypertension, we performed additional experiments to better understand the role of H_2_S during Pb-induced hypertension. Increases in oxidative stress contribute to hypertension and have been pointed as a possible mechanism underlying Pb-induced hypertension [18, 19], while H_2_S has been shown to exert antioxidant activity [79, 80]. Therefore, we sought to investigate the effects of H_2_S in oxidative stress in Pb-induced hypertension. We found that plasma from Pb group presented higher levels of MDA. As MDA is a metabolite of lipid peroxidation [81], increased MDA levels in these animals indicate higher levels of oxidative stress. In our hands, treatment of hypertensive animals with NaHS (Pb + NaHS group) blunted the increase in oxidative stress; moreover, decreased MDA levels were followed by an increase in the antioxidant status of plasma in Pb + NaHS group, as measured by the MTT assay [50]. Accordingly, previous studies have shown the role of H_2_S in reduction of oxidative stress and increases in antioxidant capacity [36, 82–84]. These results suggest that beneficial effects of H_2_S in Pb-induced hypertension may be related to H_2_S antioxidant capacity and reductions in oxidative stress.

In conclusion, we showed that increases in diastolic and mean blood pressure in human patients intoxicated with Pb may be related to decreased H_2_S plasmatic levels. Treatment with H_2_S donor blunted increases in SBP in rats and this beneficial effect of NaHS may not be related to NO. Importantly, we showed that treatment with NaHS in hypertensive animals led to vascular relaxation induced by ACh which is non-NO-mediated. Therefore, the antioxidant capacity of H_2_S may also be involved in reductions in blood pressure in Pb-induced hypertension.

## Figures and Tables

**Table 1:** KCl-induced contraction, PHE-induced contraction, and ACh-induced relaxation in thoracic aorta of Saline, NaHS, Pb, and Pb + NaHS rat.

	Aortic rings							
	+E				−E			
	Saline	NaHS	Pb	Pb + NaHS	Saline	NaHS	Pb	Pb + NaHS
KCl *R*_max⁡_, g	0.87 ± 0.06	0.84 ± 0.06	0.79 ± 0.05	0.75 ± 0.10	0.75 ± 0.11	0.83 ± 0.06	0.64 ± 0.04	0.78 ± 0.08
PHE *R*_max⁡_, g	1.05 ± 0.02	1.36 ± 0.05	0.80 ± 0.02^+^	0.84 ± 0.04^+^	1.51 ± 0.07^•^	1.79 ± 0.06^•^	1.20 ± 0.05^•+^	1.56 ± 0.06^•^
PHE pEC_50_, −log⁡M	6.84 ± 0.06	7.00 ± 0.10	6.75 ± 0.08	6.85 ± 0.12	7.50 ± 0.16^•^	7.54 ± 0.12^•^	6.95 ± 0.11	7.35 ± 0.12^•^
ACh *R*_max⁡_, %	97.79 ± 1.47	93.76 ± 2.01	93.78 ± 2.10	93.88 ± 2.12	—	—	—	—
ACh pEC_50_, −log⁡M	7.07 ± 0.20	6.99 ± 0.20	7.13 ± 0.22	7.54 ± 0.25	—	—	—	—
ACh + L-NAME *R*_max⁡_, %	20.38 ± 3.34	9.60 ± 0.70	23.08 ± 3.80	38.90 ± 4.84^*∗*+#^	—	—	—	—

Data represents mean ± SEM (*n* = 5–7). ^*∗*^*P* < 0.05 versus Saline. ^+^*P* < 0.05 versus NaHS. ^#^*P* < 0.05 versus Pb. ^•^*P* < 0.05 versus intact vessels (+E) of the same animal group.

## References

[1] Healey N. (2009). Lead toxicity, vulnerable subpopulations and emergency preparedness. *Radiation Protection Dosimetry*.

[2] Awodele O., Popoola T. D., Amadi K. C., Coker H. A. B., Akintonwa A. (2013). Traditional medicinal plants in Nigeria—Remedies or risks. *Journal of Ethnopharmacology*.

[3] Renner R. (2009). Out of plumb: when water treatment causes lead contamination. *Environmental Health Perspectives*.

[4] Álvarez-Lloret P., Lee C. M., Conti M. I., Terrizzi A. R., González-López S., Martínez M. P. (2017). Effects of chronic lead exposure on bone mineral properties in femurs of growing rats. *Toxicology*.

[5] Lee C. M., Terrizzi A. R., Bozzini C., Piñeiro A. E., Conti M. I., Martínez M. P. (2016). Chronic lead poisoning magnifies bone detrimental effects in an ovariectomized rat model of postmenopausal osteoporosis. *Experimental and Toxicologic Pathology*.

[6] Newman N. (2015). Investigation of childhood lead poisoning from parental take-home exposure from an electronic scrap recycling facility—Ohio, 2012. *MMWR Morbidity and Mortality Weekly Report*.

[7] Flores-Montoya M. G., Sobin C. (2015). Early chronic lead exposure reduces exploratory activity in young C57BL/6J mice. *Journal of Applied Toxicology*.

[8] Kosnett M. J., Wedeen R. P., Rothenberg S. J. (2006). Recommendations for medical management of adult lead exposure. *Environmental Health Perspectives*.

[9] Nascimento R. A., Mendes G., Possomato-Vieira J. S. (2015). Metalloproteinase inhibition protects against reductions in circulating adrenomedullin during lead-induced acute hypertension. *Basic & Clinical Pharmacology & Toxicology*.

[10] Glenn B. S., Stewart W. F., Links J. M., Todd A. C., Schwartz B. S. (2003). The longitudinal association of lead with blood pressure. *Epidemiology*.

[11] Alarcon W. A., State Adult Blood Lead E., Surveillance Program I. (2016). Elevated blood lead levels among employed adults—United States, 1994-2013. *MMWR Morbidity and Mortality Weekly Report*.

[12] Silveira E. A., Siman F. D. M., De Oliveira Faria T. (2014). Low-dose chronic lead exposure increases systolic arterial pressure and vascular reactivity of rat aortas. *Free Radical Biology & Medicine*.

[13] Gonçalves-Rizzi V. H., Nascimento R. A., Possomato-Vieira J. S., Dias-Junior C. A. (2016). Sodium nitrite prevents both reductions in circulating nitric oxide and hypertension in 7-day lead-treated rats. *Basic & Clinical Pharmacology & Toxicology*.

[14] Gould E. (2009). Childhood lead poisoning: conservative estimates of the social and economic benefits of lead hazard control. *Environmental Health Perspectives*.

[15] Simões M. R., Preti S. C., Azevedo B. F. (2017). Low-level chronic lead exposure impairs neural control of blood pressure and heart rate in rats. *Cardiovascular Toxicology*.

[16] Rizzi E., Castro M. M., Fernandes K. (2009). Evidence of early involvement of matrix metalloproteinase-2 in lead-induced hypertension. *Archives of Toxicology*.

[17] Dursun N., Arifoglu C., Süer C., Keskinol L. (2005). Blood pressure relationship to nitric oxide, lipid peroxidation, renal function, and renal blood flow in rats exposed to low lead levels. *Biological Trace Element Research*.

[18] Fiorim J., Ribeiro R. F., Silveira E. A. (2011). Low-level lead exposure increases systolic arterial pressure and endothelium-derived vasodilator factors in rat aortas. *PLoS ONE*.

[19] Caylak E., Aytekin M., Halifeoglu I. (2008). Antioxidant effects of methionine, *α*-lipoic acid, N-acetylcysteine and homocysteine on lead-induced oxidative stress to erythrocytes in rats. *Experimental and Toxicologic Pathology*.

[20] Zhang X., Bian J.-S. (2014). Hydrogen sulfide: a neuromodulator and neuroprotectant in the central nervous system. *ACS Chemical Neuroscience*.

[21] Feliers D., Lee H. J., Kasinath B. S. (2016). Hydrogen sulfide in renal physiology and disease. *Antioxidants & Redox Signaling*.

[22] Szabo C. (2017). Hydrogen sulfide, an enhancer of vascular nitric oxide signaling: mechanisms and implications. *American Journal of Physiology-Cell Physiology*.

[23] Jain S. K., Bull R., Rains J. L. (2010). Low levels of hydrogen sulfide in the blood of diabetes patients and streptozotocin-treated rats causes vascular inflammation?. *Antioxidants & Redox Signaling*.

[24] Cao X., Bian J.-S. (2016). The role of hydrogen sulfide in renal system. *Frontiers in Pharmacology*.

[25] Kanagy N. L., Szabo C., Papapetropoulos A. (2017). Vascular biology of hydrogen sulfide. *American Journal of Physiology-Cell Physiology*.

[26] Wallace J. L., Wang R. (2015). Hydrogen sulfide-based therapeutics: exploiting a unique but ubiquitous gasotransmitter. *Nature Reviews Drug Discovery*.

[27] Shibuya N., Tanaka M., Yoshida M. (2009). 3-Mercaptopyruvate sulfurtransferase produces hydrogen sulfide and bound sulfane sulfur in the brain. *Antioxidants & Redox Signaling*.

[28] Kimura H. (2014). The physiological role of hydrogen sulfide and beyond. *Nitric Oxide: Biology and Chemistry*.

[29] Abe K., Kimura H. (1996). The possible role of hydrogen sulfide as an endogenous neuromodulator. *The Journal of Neuroscience*.

[30] Caprnda M., Qaradakhi T., Hart J. L. (2017). H2S causes contraction and relaxation of major arteries of the rabbit. *Biomedicine & Pharmacotherapy*.

[31] Materazzi S., Zagli G., Nassini R. (2017). Vasodilator activity of hydrogen sulfide (H2S) in human mesenteric arteries. *Microvascular Research*.

[32] Hou C., Wang M., Sun C. (2016). Protective effects of hydrogen sulfide in the ageing kidney. *Oxidative Medicine and Cellular Longevity*.

[33] Spassov S. G., Donus R., Ihle P. M., Engelstaedter H., Hoetzel A., Faller S. (2017). Hydrogen sulfide prevents formation of reactive oxygen species through PI3K/Akt signaling & limits ventilator-induced lung injury. *Oxidative Medicine and Cellular Longevity*.

[34] Sun X., Wang W., Dai J. (2017). A long-term and slow-releasing hydrogen sulfide donor protects against myocardial ischemia/reperfusion injury. *Scientific Reports*.

[35] Wu W., Hou C.-L., Mu X.-P. (2017). H2S Donor NaHS Changes the Production of Endogenous H2S and NO in D-Galactose-Induced Accelerated Ageing. *Oxidative Medicine and Cellular Longevity*.

[36] Shao M., Zhuo C., Jiang R. (2017). Protective effect of hydrogen sulphide against myocardial hypertrophy in mice. *Oncotarget*.

[37] Muzaffar S., Shukla N., Bond M. (2008). Exogenous hydrogen sulfide inhibits superoxide formation, NOX-1 expression and Rac1 activity in human vascular smooth muscle cells. *Journal of Vascular Research*.

[38] Al-Magableh M. R., Kemp-Harper B. K., Ng H. H., Miller A. A., Hart J. L. (2014). Hydrogen sulfide protects endothelial nitric oxide function under conditions of acute oxidative stress in vitro. *Naunyn-Schmiedeberg's Archives of Pharmacology*.

[39] Ford A., Al-Magableh M., Gaspari T. A., Hart J. L. (2013). Chronic NaHS treatment is vasoprotective in high-fat-fed ApoE^−/−^ mice. *International Journal of Vascular Medicine*.

[40] Al-Magableh M. R., Kemp-Harper B. K., Hart J. L. (2015). Hydrogen sulfide treatment reduces blood pressure and oxidative stress in angiotensin II-induced hypertensive mice. *Hypertension Research*.

[41] Suzuki K., Olah G., Modis K. Hydrogen sulfide replacement therapy protects the vascular endothelium in hyperglycemia by preserving mitochondrial function.

[42] Wang K. Q., Ahmad S., Cai M. (2013). Dysregulation of hydrogen sulfide producing enzyme cystathionine *γ*-lyase contributes to maternal hypertension and placental abnormalities in preeclampsia. *Circulation*.

[43] Zhuo Y., Chen P.-F., Zhang A.-Z., Zhong H., Chen C.-Q., Zhu Y.-Z. (2009). Cardioprotective effect of hydrogen sulfide in ischemic reperfusion experimental rats and its influence on expression of survivin gene. *Biological & Pharmaceutical Bulletin*.

[44] Zhu M., Ren Z., Possomato-Vieira J. S., Khalil R. A. (2016). Restoring placental growth factor-soluble fms-like tyrosine kinase-1 balance reverses vascular hyper-reactivity and hypertension in pregnancy. *American Journal of Physiology-Regulatory, Integrative and Comparative Physiology*.

[45] De Baptista R. F. F., Chies A. B., De Taipeiro E. F., Cordellini S. (2014). Endothelial at_1_and at_2_ pathways in aortic responses to angiotensin II after stress and ethanol consumption in rats. *Stress*.

[46] Mosmann T. (1983). Rapid colorimetric assay for cellular growth and survival: application to proliferation and cytotoxicity assays. *Journal of Immunological Methods*.

[47] Rocha-Penha L., Caldeira-Dias M., Tanus-Santos J. E., De Carvalho Cavalli R., Sandrim V. C. (2017). Myeloperoxidase in hypertensive disorders of pregnancy and its relation with nitric oxide. *Hypertension*.

[48] Miranda K. M., Espey M. G., Wink D. A. (2001). A rapid, simple spectrophotometric method for simultaneous detection of nitrate and nitrite. *Nitric Oxide: Biology and Chemistry*.

[49] Périco L. L., Heredia-Vieira S. C., Beserra F. P. (2015). Does the gastroprotective action of a medicinal plant ensure healing effects? An integrative study of the biological effects of Serjania marginata Casar. (Sapindaceae) in rats. *Journal of Ethnopharmacology*.

[50] Medina L. O., Veloso C. A., de Abreu Borges É. (2007). Determination of the antioxidant status of plasma from type 2 diabetic patients. *Diabetes Research and Clinical Practice*.

[51] Lopes A. C. B. D. A., Silbergeld E. K., Navas-Acien A. (2017). Association between blood lead and blood pressure: a population-based study in Brazilian adults. *Environmental Health: A Global Access Science Source*.

[52] Vupputuri S., He J., Muntner P., Bazzano L. A., Whelton P. K., Batuman V. (2003). Blood lead level is associated with elevated blood pressure in blacks. *Hypertension*.

[53] Cheng Y., Schwartz J., Sparrow D., Aro A., Weiss S. T., Hu H. (2001). Bone lead and blood lead levels in relation to baseline blood pressure and the prospective development of hypertension. The normative aging study. *American Journal of Epidemiology*.

[54] Barbosa F., Gerlach R. F., Tanus-Santos J. E. (2006). Matrix metalloproteinase-9 activity in plasma correlates with plasma and whole blood lead concentrations. *Basic & Clinical Pharmacology & Toxicology*.

[55] Shakir S. K., Azizullah A., Murad W. (2017). Toxic Metal Pollution in Pakistan and Its Possible Risks to Public Health. *Reviews of Environmental Contamination and Toxicology*.

[56] van Goor H., van den Born J. C., Hillebrands J., Joles J. A. (2016). Hydrogen sulfide in hypertension. *Current Opinion in Nephrology and Hypertension*.

[57] Greaney J. L., Kutz J. L., Shank S. W., Jandu S., Santhanam L., Alexander L. M. (2017). Impaired hydrogen sulfide-mediated vasodilation contributes to microvascular endothelial dysfunction in hypertensive adults. *Hypertension*.

[58] Cacanyiova S., Berenyiova A., Kristek F., Drobna M., Ondrias K., Grman M. (2016). The adaptive role of nitric oxide and hydrogen Sulphide in vasoactive responses of thoracic aorta is triggered already in young spontaneously hypertensive rats. *Journal of Physiology and Pharmacology*.

[59] Zhang Y., Weiner J. H. (2014). A simple semi-quantitative in vivo method using H_2_S detection to monitor sulfide metabolizing enzymes. *BioTechniques*.

[60] Dieter M. P., Matthews H. B., Jeffcoat R. A., Moseman R. F. (1993). Comparison of lead bioavailability in f344 rats fed lead acetate, lead oxide, lead sulfide, or lead ore concentrate from skagway, alaska. *Journal of Toxicology and Environmental Health*.

[61] Greiner R., Pálinkás Z., Bäsell K. (2013). Polysulfides link H_2_S to protein thiol oxidation. *Antioxidants & Redox Signaling*.

[62] Huang Y., Yu F., Wang J., Chen L. (2016). Near-infrared fluorescence probe for in situ detection of superoxide anion and hydrogen polysulfides in mitochondrial oxidative stress. *Analytical Chemistry*.

[63] Nagy P., Winterbourn C. C. (2010). Rapid reaction of hydrogen sulfide with the neutrophil oxidant hypochlorous acid to generate polysulfides. *Chemical Research in Toxicology*.

[64] Nagy P., Pálinkás Z., Nagy A., Budai B., Tóth I., Vasas A. (2014). Chemical aspects of hydrogen sulfide measurements in physiological samples. *Biochimica et Biophysica Acta*.

[65] Olson K. R. (2018). H_2_S and polysulfide metabolism: conventional and unconventional pathways. *Biochemical Pharmacology*.

[66] Murphy J. G., Herrington J. N., Granger J. P., Khalil R. A. (2003). Enhanced [Ca^2+^]i in renal arterial smooth muscle cells of pregnant rats with reduced uterine perfusion pressure. *American Journal of Physiology-Heart and Circulatory Physiology*.

[67] Ali M. Y., Ping C. Y., Mok Y. Y. P. (2006). Regulation of vascular nitric oxide in vitro and in vivo; a new role for endogenous hydrogen sulphide?. *British Journal of Pharmacology*.

[68] Whiteman M., Li L., Kostetski I. (2006). Evidence for the formation of a novel nitrosothiol from the gaseous mediators nitric oxide and hydrogen sulphide. *Biochemical and Biophysical Research Communications*.

[69] Liu Y.-H., Bian J.-S. (2010). Bicarbonate-dependent effect of hydrogen sulfide on vascular contractility in rat aortic rings. *American Journal of Physiology-Cell Physiology*.

[70] Jia J. L., Liu Y.-H., Khin E. S. W., Bian J.-S. (2008). Vasoconstrictive effect of hydrogen sulfide involves downregulation of cAMP in vascular smooth muscle cells. *American Journal of Physiology-Cell Physiology*.

[71] Hosoki R., Matsuki N., Kimura H. (1997). The possible role of hydrogen sulfide as an endogenous smooth muscle relaxant in synergy with nitric oxide. *Biochemical and Biophysical Research Communications*.

[72] Xiao L., Dong J.-H., Jin S. (2016). Hydrogen sulfide improves endothelial dysfunction via downregulating BMP4/COX-2 pathway in rats with hypertension. *Oxidative Medicine and Cellular Longevity*.

[73] Zhao W., Zhang J., Lu Y., Wang R. (2001). The vasorelaxant effect of H_2_S as a novel endogenous gaseous K_ATP_ channel opener. *EMBO Journal*.

[74] Zhao W., Wang R. (2002). H_2_S-induced vasorelaxation and underlying cellular and molecular mechanisms. *American Journal of Physiology-Heart and Circulatory Physiology*.

[75] Aydinoglu F., Dalkir F. T., Demirbag H. O., Ogulener N. (2017). The interaction of L-cysteine/H_2_S pathway and muscarinic acetylcholine receptors (mAChRs) in mouse corpus cavernosum. *Nitric Oxide: Biology and Chemistry*.

[76] Wang Y. F., Mainali P., Tang C. S. (2008). Effects of nitric oxide and hydrogen sulfide on the relaxation of pulmonary arteries in rats. *Chinese Medical Journal*.

[77] Chen P.-H., Fu Y.-S., Wang Y.-M., Yang K.-H., Wang D. L., Huang B. (2014). Hydrogen sulfide increases nitric oxide production and subsequent S-nitrosylation in endothelial cells. *The Scientific World Journal*.

[78] Huang B., Chen C.-T., Chen C.-S., Wang Y.-M., Hsieh H.-J., Wang D. L. (2015). Laminar shear flow increases hydrogen sulfide and activates a nitric oxide producing signaling cascade in endothelial cells. *Biochemical and Biophysical Research Communications*.

[79] Zhou X., Zhao L., Mao J., Huang J., Chen J. (2015). Antioxidant effects of hydrogen sulfide on left ventricular remodeling in smoking rats are mediated via PI3K/Akt-dependent activation of Nrf2. *Toxicological Sciences*.

[80] Aghagolzadeh P., Radpour R., Bachtler M. (2017). Hydrogen sulfide attenuates calcification of vascular smooth muscle cells via KEAP1/NRF2/NQO1 activation. *Atherosclerosis*.

[81] Ohkawa H., Ohishi N., Yagi K. (1979). Assay for lipid peroxides in animal tissues by thiobarbituric acid reaction. *Analytical Biochemistry*.

[82] Chen X., Zhao X., Cai H. (2017). The role of sodium hydrosulfide in attenuating the aging process via PI3K/AKT and CaMKK*β*/AMPK pathways. *Redox Biology*.

[83] Yang R., Jia Q., Liu X.-F., Wang Y.-Y., Ma S.-F. (2017). Effects of hydrogen sulfide on inducible nitric oxide synthase activity and expression of cardiomyocytes in diabetic rats. *Molecular Medicine Reports*.

[84] Xie L., Yu S., Yang K., Li C., Liang Y. (2017). Hydrogen sulfide inhibits autophagic neuronal cell death by reducing oxidative stress in spinal cord ischemia reperfusion injury. *Oxidative Medicine and Cellular Longevity*.

